# Clerical Exposures

**Published:** 1858-01

**Authors:** 


### CLERICAL EXPOSURES.

A STERN and honorable sense of duty has led many a self-sacrificing clergyman and physician to encounter exposures which have laid them in the tomb; and many a martyr to professional obligation sleeps in his lonely and forgotten grave.

An active, talented, and efficient clergyman, from the far West, writes: "Three weeks ago, I overdid myself in walking, caught a cold, preached with the cold on me; rode out immediately afterwards to see a dying man, took a fresh cold, which settled on my lungs, coughed tremendously for a few days, had asthmatic symptoms, but in eight or ten days all disappeared, and I think the lungs are free from all disease." At the same time, the fore part of the letter complained, "On preaching days I experience a sensation of relaxation in throat and whole body, down to fingers and toes, huskiness of voice, and a slight soreness about the hollow at the bottom of the neck."

Riding on horseback immediately after a public address, in damp or rainy weather, or windy weather, even in summer time, is enough to fasten a fatal disease on any man of ordinary health. Public men must decide for themselves how far they are called upon to take risks, with chances so largely against them.

As to preaching with the hoarseness of a fresh cold upon him, no man is justifiable under any circumstances short of threatened life.

After speaking in weathers above named, persons should remain in the house at least twenty minutes, then button up, and keep the nose and mouth well veiled.

